# Animalistic slurs increase harm by changing perceptions of social desirability

**DOI:** 10.1098/rsos.230203

**Published:** 2023-07-12

**Authors:** Florence E. Enock, Harriet Over

**Affiliations:** ^1^ The Alan Turing Institute, London, UK; ^2^ University of York, York, North Yorkshire, UK

**Keywords:** dehumanization, dehumanizing language, propaganda, intergroup harm, social cognition

## Abstract

In propaganda and hate speech, target groups are often compared to dangerous and disgusting animals. Exposure to these animalistic slurs is thought to increase endorsement of intergroup harm but the mechanism by which this happens remains unclear. Across two pre-registered and highly powered studies, we examined how animalistic language influences the cultural transmission of beliefs about target groups. In line with previous work, we found that describing a novel political group with animalistic slurs increased the extent to which participants endorsed harm towards them. Importantly, reading animalistic slurs did not influence the extent to which participants believed the target group possessed uniquely human qualities. Rather, the animalistic slurs influenced endorsement of harm by making the target group appear more undesirable. These findings offer a novel perspective into the nature of dehumanization and new insights into how hate speech functions.

**Statement of relevance**

Propaganda and hate speech regularly incorporate references to the victim group as dangerous and disgusting animals, such as rats and cockroaches. These animalistic slurs lead to further intergroup harm. Given the prevalence of dangerous online hate speech, accurately understanding its societal impact on attitudes and behaviours is crucial. One possibility is that animalistic slurs lead the target group to be viewed as possessing fewer uniquely human qualities. Alternatively, animalistic slurs may serve as metaphors which lead to negative perceptions of the outgroup. Despite receiving theoretical attention across the social sciences, the function of dehumanizing language in influencing beliefs about, and behaviour towards, target groups has received scant empirical attention. Across two experiments, we show that animalistic slurs increased harm by leading the target group to be perceived as undesirable. Animalistic slurs did not influence the extent to which the target group was seen to possess uniquely human character traits and emotions.

## 1. Introduction

Propaganda and hate speech regularly incorporate references to victim groups as dangerous and disgusting animals. For example, in Nazi Germany, anti-Semitic propaganda compared Jewish people to rats, lice and parasites. Before and during the genocide in Rwanda in 1994, Hutu propaganda referred to Tutsis as snakes and cockroaches [1–4]. Instances such as these are not limited to the past. In contemporary Hungary, Roma people have been described as animals, ‘not fit to live among people’ [5], while undocumented migrants have been referred to as ‘swarms’ and ‘animals’ by political leaders in the UK and in the USA [6,7].

Researchers have sought to analyse propaganda and hate speech in order to help understand the psychological mechanisms which contribute to intergroup harm. According to arguments across psychology, neuroscience, philosophy, history and sociology, a psychological process of dehumanization often lies at the heart of intergroup harm. When outgroup members are blatantly dehumanized, they are thought to be viewed as less than human creatures and explicitly compared to non-human animals [2,8]. When outgroup members are subtly dehumanized, they are thought to be denied uniquely human qualities such as refinement, rationality and civility [9,10] as well as uniquely human emotions such as pride, nostalgia and regret [11,12]. When a group are perceived to be less than human [2,13], or less human than the ingroup [9,14], it is thought that natural inhibitions against causing them harm are eroded leading, in extreme cases, to genocide and torture [14–16]. In more subtle cases, dehumanization is hypothesized to lead to increases in punishment and reductions in prosocial behaviour [8,17,18].

There is a growing debate in the literature regarding how the language used by propagandists relates to their underlying beliefs about the victim group. In line with theorizing on dehumanization, one possibility is that animalistic slurs often reflect propagandists' literal belief that victim groups are less than human. For example, Smith argues that ‘…dehumanizers are not just slinging animalistic metaphors at a vulnerable group… They sincerely believe that those whom they persecute are less than human.’ [3, p. 28]. Haslam and Loughnan note that when individuals are animalistically dehumanized, ‘they should be seen as lacking refinement, self-control, intelligence and rationality, and subtly or overtly likened to animals' [10, p. 403].

Such language may not always reflect the literal beliefs of propagandists, however. Animalistic slurs can also be used cynically to create hatred and division regardless of whether or not the propagandists themselves believe the victim group to be less than human or less human than the ingroup [19–22]. On this reading, animalistic slurs are one strategy among several that propagandists use to try to maximize antipathy towards their victim group. Broadly supporting this view, the historical record shows that comparisons to non-human entities are not reserved for victim groups [22]. Animalistic metaphors can be used to highlight the supposedly desirable qualities of the ingroup and undesirable and/or threatening qualities of the outgroup. For example, in notorious anti-Semitic Nazi propaganda, *Der Giftpilz (The Poisonous Mushroom)*, both ingroup and outgroup members are compared to non-human entities: ‘There are good mushrooms and there are good people. There are poisonous, bad mushrooms and there are bad people’. In addition, animalistic slurs often appear in propaganda alongside references to the victim group as enemies, criminals and traitors—terms that make most sense when applied to humans [19,21–23]. These terms may all serve to increase antipathy towards the victim group.

To date, there has been considerably less investigation of how the writing of propagandists in general, and their use of animalistic slurs in particular, relates to the beliefs of their audience. While the beliefs and intentions of propagandists are not easily accessible to experimental investigation, the effects of hate speech on attitudes and behaviour can be measured in laboratory-based settings. It is widely hypothesized that describing a victim group with animalistic slurs influences the attitudes and behaviour of other ingroup members, encouraging them to acquiesce to, or even actively participate in, further harming the target group [4,24]. Broadly supporting this claim, laboratory-based research has shown positive correlations between animalistic descriptions and endorsement of intergroup harm [8,25].

To date, much of the research investigating the connection between animalistic slurs and intergroup harm has been theoretical or correlational. One exception is an experimental study by Bandura and colleagues in which target group members were either described in terms of an animalistic slur (an animalistic rotten bunch) or in more positive human terms (perceptive and understanding). Targets described in animalistic language were shown more aggression [26]. However, it remains unclear whether the observed difference between conditions was driven by the animalistic description in the dehumanized condition or the more desirable character traits referenced in the control condition. Our first goal, therefore, was to confirm that reading animalistic slurs increases endorsement of harm when the sentences are otherwise matched in content.

Our principal question of interest, however, was to understand how animalistic comparisons influence how a group is perceived. One reasonable possibility is that when people read animalistic slurs, they come to view the target group as less than human [2,3,13] or as lacking uniquely human qualities [9,12]. These dehumanized perceptions may then place the group at increased risk of harm [26–28]. A second possibility, drawing on recent critiques of the dehumanization literature, is that when people read animalistic slurs, they come to believe that the target group has socially undesirable characteristics, while retaining an understanding of its members as human [22,29–31]. Under this account, it is increased antipathy that places the target group at greater risk of harm.

To test these questions, we described novel political groups with and without animalistic slurs and measured the effects of these slurs on participants' beliefs about each group. To do this, we drew on two of the most prominent theories of dehumanization from social psychology, the dual model [9], according to which outgroup members may be dehumanized by being denied uniquely human character traits, and infrahumanization theory, according to which outgroup members may be dehumanized by being denied uniquely human emotions [11,12]. We compared how animalistic slurs influence participants’ attributions of traits and emotions that are unique to humans and those that are shared with other animals. Crucially, we also compared attributions of traits and emotions that are socially desirable and socially undesirable.

If animalistic slurs induce dehumanizing beliefs in an audience, then according to operationalizations of dehumanization from social psychology, which focus on the attribution of uniquely human traits and emotions [9,11,12], target groups likened to animals may be perceived as possessing uniquely human qualities to a lesser extent than those not compared to animals. If, as we predicted, animalistic slurs induce beliefs about socially undesirable characteristics, then groups compared to animals should be perceived as possessing socially desirable qualities to a lesser extent but socially undesirable qualities to a greater extent than those not compared to animals. It is possible, of course, that both processes will operate in parallel. We designed our studies such that it was possible to examine whether animalistic slurs change perceptions of humanness, social desirability, or both simultaneously.

In each study, we also measured how changes in trait and emotion attributions are connected to endorsement of harm. One possibility is that animalistic slurs will be associated with greater endorsement of harm through influencing the perceived humanness of the target group, measured by the extent to which the target group is seen to possess uniquely human traits and emotions. Another possibility, and the one that we favoured, is that animalistic slurs will be associated with greater endorsement of harm through influencing the perceived social desirability of the target group, measured by the extent to which the target group is viewed as possessing socially desirable and undesirable qualities.

### 1.1. Data collection and open science

All data collection took place online and studies were created and administered using Qualtrics (https://www.qualtrics.com). Participants were recruited through Prolific (https://www.prolific.co) and different participants took part in each study. Informed consent was obtained at the start of each session according to approved ethical procedures. Participants were compensated at an approximate rate of £7.50 per hour. Both studies reported in this article were pre-registered and the data is available open access. Link to pre-registration documents and raw data for each study can be found at: https://osf.io/4pvs3/.

## 2. Study 1: animalistic slurs, trait attributions and links to harm

We measured whether describing a novel political group with animalistic slurs changed the extent to which they were viewed as having uniquely human qualities, the extent to which they are viewed as having undesirable qualities, or both. In order to do this, we drew on the dual model of dehumanization according to which dehumanization can be operationalized as a tendency to deny a group uniquely human character traits [9].

We also measured how any change in character perception relates to harm. One possibility is that reading animalistic slurs will lead participants to view the target group as less human, here shown in a denial of uniquely human traits. A dehumanized perception of the target group will then explain the connection between animalistic slurs and harm. Another possibility, and one that we favoured, is that reading animalistic slurs about a group will lead participants to view them as less socially desirable in character, shown in a denial of socially desirable traits and an increase in attribution of socially undesirable traits. According to our hypothesis, this negative perception of the target group will explain the connection between animalistic slurs and endorsement of harm.

### 2.1. Methods

#### 2.1.1. Participants

A total of 208 participants completed the study. For the 2 × 2 × 2 within subjects ANOVA, a sample size analysis found a minimum N of 207 to be necessary to detect effects of interest with a medium effect size $\left(\eta_{p}^{2}=0.06\right)$, α of 0.05 and power of 0.95. For even counterbalancing across the two versions, we included 208 participants. A sensitivity analysis showed that with this sample size, we could detect a small to medium effect size (*f*^2^ = 0.09) with power of 0.95 in each of the four regression analyses with a corrected α of 0.01 (0.05/four tests). The power analyses were conducted in G*power 3.1.

Participants were all 18 or over, fluent in English, and UK nationals living in the UK. In line with our pre-registration, we excluded and replaced nine participants who failed the attention check. Of the final sample, 145 participants identified as female, 61 as male and two as non-binary. Participants were aged from 18 to 72 with a mean age of 32.6 (s.d. = 11.5).

#### 2.1.2. Design

We employed a within-subjects design in which participants each read about two fictional groups, one described in sentences likening them to non-human animals, and one described in similar (though not identical) sentences but without animalistic comparisons. Participants then indicated the extent to which they believed each group to possess a number of traits, designed to measure perceptions of humanness and of social desirability as orthogonal constructs [29,30]. We tested whether animalistic slurs altered perceptions of humanness, of social desirability, or both. Participants also indicated the extent to which they would endorse harm to both groups. We tested whether animalistic slurs led to greater harm endorsement. We also tested whether greater harm endorsement for those described in animalistic terms was driven by changes in perceptions of humanness, changes in perceptions of social desirability, or both.

#### 2.1.3. Materials

##### 2.1.3.1. Experimental manipulation—animalistic and control descriptions

Participants each read about members of two fictional political parties (the ‘MRP’ and the ‘NLP’), who were each described in a series of 10 vox pop-style quotes in which unnamed individuals expressed reluctance to vote for the parties. One political party was described in sentences that contained comparisons to non-human animals (animalistic condition) and the other party was described in broadly matched (but not identical) sentences without comparisons to non-human animals (control condition). Participants read about the two groups in two separate sequential experimental blocks, with one block thus corresponding to each of the experimental conditions, animalistic and control.

The sentences used for the language manipulation are shown in table 1. We created two sets of similar sentences which could describe either party and which could represent the animalistic or control condition. For example, if a participant read sentences from set 1 about the MRP and including animalistic terms, they would correspondingly see sentences from set 2 about the NLP and excluding animalistic terms (control). If a participant read sentences from set 1 about the MRP and excluding animalistic terms, they would correspondingly see sentences from set 2 about the NLP and including animalistic terms.

**Table 1.** Sentence sets for the language manipulation. Each of the sentence sets could describe either party and each could represent either the animalistic or control condition. The non-human comparisons added to create the animalistic condition are shown in italics in each.

	sentence set 1	sentence set 2
1	I believe history has shown that we should not vote for the [MRP/NLP] *(, they are vermin)*.	I don't believe an [MRP/NLP] government has ever made a positive contribution *(, they are rats)*.
2	I tend to think the new campaign of *(those rats)* the [MRP/NLP] is untenable.	I think *(those snakes)* the [MRP/NLP] are driving forward a shaky campaign.
3 (filler)	My sister supports the [MRP/NLP] so we have a lot of debates at the moment in our family!	I feel there are so many things to worry about as well as the election at the moment.
4	I feel we would be better off without the [MRP/NLP] in power. *(They are snakes.)*	I don't believe there are any benefits to an [MRP/NLP] leadership. *(They are cockroaches.)*
5	I would prefer not to live in a country run by *(the cockroaches that are)* the [MRP/NLP].	My family and I would prefer not to be governed by *(the vermin that are)* the [MRP/NLP].
6 (filler)	To be honest I just want this election to be over, it has divided my workplace.	For our children's sake, I just want good resources for education.
7	I don't believe *(the apes that are)* the [MRP/NLP] are the leaders that our country deserves.	I struggle to see the value to our country if *(the pigs that are)* the [MRP/NLP] become our leaders.
8	I have already planned big celebrations with my family for if we beat *(those dogs)* the [MRP/NLP]!	I can't even imagine the relief I'll feel if we can defeat *(those apes)* the [MRP/NLP]!
9 (filler)	I don't think such a thing exists as a perfect party with perfect leaders.	I just wish our country could become more united.
10	I don't believe the policies in the [MRP's/NLP's] manifesto will make the country better *(, they are such pigs).*	I don't think the manifesto of the [MRP/NLP] will be a force for positive progress *(, they are dogs)*.

In the animalistic condition, seven of these sentences contained references to non-human animals while three sentences served as more neutral filler items. The animal terms were: apes, cockroaches, dogs, pigs, rats, snakes, vermin. We chose these terms because they are commonly found in propaganda and hate speech and said to be paradigmatic of dehumanization in language [1,2,4,32]. In the control condition, none of the sentences contained animal terms.

##### 2.1.3.2. Counterbalancing the language manipulation

Each of the experimental conditions (animalistic and control) could be represented in either sentence set 1 or 2, by descriptions either of the ‘MRP’ or the ‘NLP’, and could appear either first or second.

For half of the participants the MRP were compared to non-human animals and for the other half the NLP were compared to non-human animals. For half of the participants sentence set 1 included the comparisons to non-human animals and, for the other half, sentence set 2 included the comparisons to non-human animals. For half of the participants the animalistic condition appeared first and for half the control condition appeared first. This resulted in eight versions of the language manipulation, counterbalanced evenly across participants (see electronic supplementary material, table S1).

##### 2.1.3.3. Dependent variable 1—trait attributions

To measure dehumanization as operationalized by the dual model [9], participants rated the extent to which they thought each target group possessed a series of 20 different character traits. The traits were taken directly from related prior work on trait-based dehumanization ([29,30], studies 2 and 3) and were previously chosen from a pretest to represent four categories of interest: uniquely human and desirable, uniquely human and undesirable, shared with other animals and desirable, and shared with other animals and undesirable. The trait categories thus took a 2 × 2 structure (humanness: uniquely human/shared with other animals × desirability: desirable/undesirable) (table 2). See electronic supplementary material for more about selection of traits.

**Table 2.** Trait items included for each condition in Study 1.

	uniquely human	shared with animals
desirable	civilized	calm
	cultured	curious
	knowledgeable	energetic
	open-minded	genuine
	sophisticated	trusting
undesirable	arrogant	inflexible
	bitter	stupid
	controlling	uncultured
	corrupt	unrefined
	superficial	unsophisticated

Participants indicated the extent to which they thought each trait word was typical of the average member of each party, the MRP and the NLP, across two blocks (one for each group condition). For each item, participants indicated their response on a sliding scale from *Not at all* (0) to *Very much so* (100), with the midpoint *Somewhat* (50), though they could not see the numbers. For example, a block could begin:

*Using the sliding scales below, please indicate the extent to which you think each trait word is typical of the average member of the MRP*.

Then, participants would respond to each item, such as ‘Members of the MRP are typically cultured’, using the unmarked sliding scale. The 20 items within each block were randomized. One attention check per block was included approximately halfway through (e.g. ‘Members of the MRP are: Please indicate “not at all”’).

Following our pre-registered plan, scores for each type of trait were obtained by calculating the mean of the five traits within that category for each participant. For example, a participant's attribution of uniquely human desirable traits was the mean of their ratings for cultured, civilized, knowledgeable, open-minded and sophisticated.

##### 2.1.3.4. Dependent variable 2—harm endorsement

We operationalized harm as behaviours that could have an adverse effect on the group members in question, such as imposing funding restrictions and signing a petition for the party to be dissolved. While theoretical work studying the role of dehumanizing language in intergroup harm often focuses on physical forms of violence [1,4,27,33], the empirical literature in social psychology also considers links between dehumanization and non-physical harm such as criminal justice sentencing, visa allocation for immigration, and petitioning against particular ideological groups [8,17]. We chose to focus on the endorsement of non-physical behaviours which could have an adverse effect on the target group because the targets were members of novel political groups with whom participants had no opportunity for direct contact.

Four items measured the endorsement of harm towards each group. These were:

1. How likely would you be to sign a petition calling for the [MRP/NLP] to be dissolved?
2. How likely would you be to attend a protest against the [MRP/NLP]?
3. How severe do you think the limitations on [MRP/NLP] funding should be?
4. How severe do you think restrictions on [MRP/NLP] social media use should be?

There were two harm endorsement blocks, one for each group condition. The four items within each block were presented in a random order and one attention check per block was included approximately halfway through (e.g. ‘How severe do you think: please indicate “Not at all severe”’). For each item, participants indicated their response on an unmarked sliding scale from *Not at all [likely/severe]* (0) to *Extremely [likely/severe]* (100).

The four-item harm endorsement scales showed good internal reliability (*α* = 0.86 for animalistic condition; *α* = 0.83 for control). The items were combined using the mean into a single harm endorsement score for each target group.

#### 2.1.4. Procedure

Participants were informed that the study was designed to help us understand the ways in which people ascribe character traits to different groups of individuals and make behavioural decisions about them. Participants were instructed they would read some short sentences about two unknown political groups and then respond to some questions about the typical character traits of members of each group and about some hypothetical behaviours towards those groups. Once informed consent was obtained, brief demographic (age, country of residence, English fluency, gender and nationality) questions were asked. Screening for eligibility was through Prolific and we also confirmed participants met the requirements within the demographic questions.

On beginning the main study, participants were first told that they would read a short fictional story and were asked to pay close attention. They then read:

*In a Southern European country, political campaigns are underway for the next general election, due to be held in about a month. The vote is predicted to be extremely close between two of the country's main parties: The NLP and the MRP*.

*To find out about voters' feelings towards the campaigns, a politically independent national newspaper gathered some vox pops from several members of the public in the country's capital*.

*You will see some quotes extracted from these vox pops. The first set of quotes that you will see are about some of the residents' feelings towards the [‘MRP'/NLP]*.

Following the introduction, participants read the first set of sentences. Sentences were presented individually and participants could only move on from each sentence after 3 s to ensure they had read it in full (there was no maximum time that participants could spend on each sentence). Whether the first sentences described the ‘MRP’ or the ‘NLP’ and whether they represented the animalistic or the control condition depended on which counterbalanced version the participant was assigned to (see above). Participants then completed an unrelated filler task for 1 min. After the filler task, participants completed the trait attribution scale in which they considered the typical characteristics of the party referred to in the first sentence set. Next, participants completed the harm endorsement scale with reference to the same party.

Once participants had completed the harm endorsement scale for the first party, they were informed that they were halfway through the study and that they would next see some more quotes extracted from the vox pops. They were informed that the next set of quotes would be about the other party (i.e. if they saw quotes about the ‘MRP’ first, they would now see quotes about the ‘NLP’). Participants then read the second set of sentences. As for the first set, each sentence was presented individually and shown on the screen for at least 3 s before it was possible to move to the next. Participants then completed another unrelated filler task for 1 min. After the filler, participants completed trait attributions and then the harm endorsement scale for the party referred to in the second sentence set.

On finishing the main study questions, participants were asked an optional question about their ethnicity and then had the opportunity to provide written feedback about the study. Finally, participants were debriefed and redirected back to Prolific for payment. Most participants took under 12 min to complete the study.

### 2.2. Results

#### 2.2.1. The influence of animalistic slurs on perceptions of humanity and social desirability

A 2(condition: animalistic control) × 2(trait humanness: uniquely human/shared with other animals) × 2 (trait desirability: desirable/undesirable) within-subjects ANOVA measured the effect of animalistic slurs on trait attributions. If animalistic slurs lead to representations of the target group as less human, then an interaction between condition and humanness should show ratings of uniquely human traits to be lower in the animalistic than in the control condition. If animalistic slurs lead to representations of the target group as undesirable in character, then an interaction between condition and social desirability should show ratings of desirable traits to be lower in the animalistic than in the control condition, and ratings of undesirable traits to be higher in the animalistic than in the control condition.

There was no main effect of condition on trait attributions, *F*_1,207_ = 2.03, *p* = 0.156, $\eta_{p}^{2}=0.010$, but there was a main effect of humanness, *F*_1,207_ = 38.65, *p* < 0.001, $\eta_{p}^{2}=0.157$ and of trait desirability, *F*_1,207_ = 258.98, *p* < 0.001, $\eta_{p}^{2}=0.556$. Ratings were higher overall for undesirable than for desirable traits, and for uniquely human than for shared traits.

Importantly, there was a three-way interaction between condition, humanness and desirability *F*_1,207_ = 10.56, *p* = 0.001, $\eta_{p}^{2}=0.049$. This was broken down using simple pairwise comparisons between the animalistic and control condition for each of the four trait conditions. In line with our predictions, and counter to the hypothesis drawn from the dehumanization literature, the target group was attributed desirable uniquely human traits and desirable shared traits to a lesser extent in the animalistic condition than in the control condition (both *p*s < 0.001). Further in line with our predictions, the target group were attributed undesirable uniquely human traits and undesirable shared traits to a greater extent in the animalistic condition than the control condition (both *ps* < 0.001).

Measuring whether animalistic slurs alter perceptions of social desirability, there was an interaction between condition and trait desirability, *F*_1,207_ = 89.54, *p* < 0.001, $\eta_{p}^{2}=0.302$. Ratings were higher for the control condition than the animalistic condition for desirable traits, but higher for the animalistic condition than the control condition for undesirable traits (all *ps* < 0.001).

Measuring whether animalistic slurs alter perceptions of humanness, there was an interaction between condition and trait humanness, *F*_1,207_ = 14.41, *p* < 0.001, $\eta_{p}^{2}=0.065$. However, this interaction did not reflect lower attribution of uniquely human traits to targets described using animalistic language. For uniquely human traits, there was no significant difference in ratings between the two conditions (*p* = 0.085). For traits shared with other animals, ratings were overall higher for the animalistic condition than for the control condition (*p* = 0.001).

The theoretically uninteresting interaction between trait humanness and trait desirability was also significant, *F*_1,207_ = 57.22, *p* < 0.001, $\eta_{p}^{2}=0.217$. There was no difference in ratings for uniquely human and shared desirable traits (*p* = 0.132). However, ratings were higher overall for uniquely human undesirable traits than for shared undesirable traits (*p* < 0.001). Ratings were higher for undesirable than desirable items across both levels of humanness (both *ps* < 0.001).

**Figure 1.**
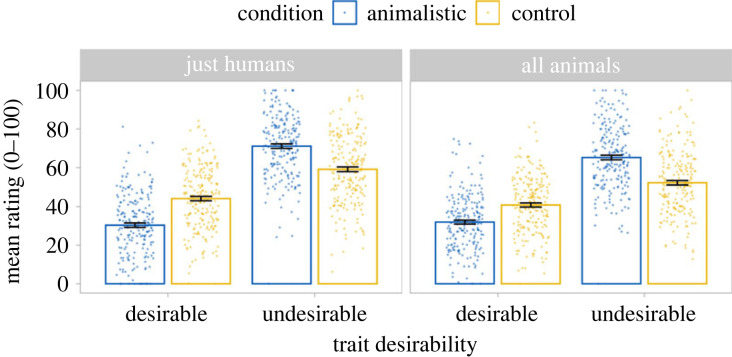
Mean trait attributions by target group, humanness and desirability for Study 1. Mean ratings were higher for the control than the animalistic condition on desirable traits but higher for the animalistic condition than the control on undesirable traits. This was true for both traits perceived as uniquely human and for those shared with other species. Error bars represent standard errors.

Figure 1 shows mean trait attributions for the animalistic and control conditions by trait humanness and desirability. Electronic supplementary material, figure S1 shows these trait attributions broken down by items within each condition.

#### 2.2.2. The influence of animalistic slurs on harm endorsement

We measured whether participants were more likely to endorse harming the target group following exposure to animalistic slurs. In order to do this, we compared harm endorsement in the animalistic condition to harm endorsement in the control condition using a within subjects *t*-test. We predicted that endorsement of harm would be higher in the animalistic condition than in the control condition. Confirming previous theorizing, participants expressed greater harm endorsement towards the target group in the animalistic condition (*M* = 58.2, s.e. = 1.63) than in the control condition (*M* = 47.3, s.e. = 1.50), *t*_207_ = 7.45, *p* < 0.001, *d* = 0.52.

#### 2.2.3. The relationship between animalistic slurs, trait attribution and harm endorsement

We tested the mechanism by which describing a group with animalistic slurs influenced endorsement of harm towards that group. If comparisons to animals increase a group's risk of harm through reducing perceptions of their humanity, then we would expect an increase in harm for the animalistic condition relative to the control to be predicted by a decrease in uniquely human trait attributions and/or an increase in attributions of traits shared with other animals for the animalistic condition relative to the control.

If comparisons to non-human animals increase a group's risk of harm through changing perceptions of their social desirability, then we would expect an increase in harm for the animalistic condition relative to the control to be predicted by a decrease in desirable trait attributions and/or an increase in undesirable trait attributions.

Four linear regressions tested whether an increase in harm endorsement towards targets in the animalistic condition relative to the control was predicted by (1) a decrease in uniquely human trait attributions; (2) an increase in attributions of traits shared with other animals; (3) a decrease in desirable trait attributions; (4) an increase in undesirable trait attributions.

#### 2.2.4. Creating relative harm endorsement scores (outcome variable)

To create a relative harm score representing the difference in harm endorsement for the animalistic compared with the control condition, we subtracted each participant's harm endorsement score for the control condition from their harm endorsement score for the animalistic condition.

#### 2.2.5. Creating relative trait attribution scores (predictor variables)

To create a relative humanness attribution score representing the difference in attribution of uniquely human traits for the animalistic condition compared with the control, we created the mean of all uniquely human traits for each participant for each condition, and we then subtracted the mean uniquely human trait attribution score for the control condition from the mean uniquely human trait attribution score for animalistic condition. This gave a score showing the difference in humanness attribution between the animalistic and control conditions. Positive numbers here represent a tendency for participants to believe targets compared to animals possess uniquely human qualities to a greater extent than the control, negative numbers indicate the reverse.

We used the same method to create a relative score representing the difference in attribution of traits shared with other animals for the animalistic condition compared with the control, where positive numbers represent a tendency for participants to believe targets compared to animals possess traits shared with other animals to a greater extent than the control, and negative numbers indicate the reverse.

We created a relative score representing the difference in attribution of desirable traits for the animalistic condition compared with the control by creating a mean of the desirable traits for each participant for each condition, and then subtracting the mean desirable trait attribution score for the control condition from the mean desirable trait attribution score for animalistic condition. Positive numbers here represent a tendency for participants to believe targets compared to animals possess desirable qualities to a greater extent than the control, negative numbers indicate the reverse. We used the same method to create a relative score representing the difference in attribution of undesirable traits for the animalistic condition compared with the control, where positive numbers represent a tendency for participants to believe targets compared to animals possess undesirable traits to a greater extent than the control, and negative numbers indicate the reverse.

We used these relative scores to test the predictions using four linear regressions:

1. Is the increase in harm for the animalistic condition relative to the control predicted by a decrease in uniquely human trait attributions for the animalistic condition relative to the control?
2. Is the increase in harm for the animalistic condition relative to the control predicted by an increase in trait attributions shared with other animals for the animalistic condition relative to the control?
3. Is the increase in harm for the animalistic condition relative to the control predicted by a decrease in desirable trait attributions for the animalistic condition relative to the control?
4. Is the increase in harm for the animalistic condition relative to the control predicted by an increase in undesirable trait attributions for the animalistic condition relative to the control?

A strength of this approach is that we could plausibly observe effects of both dehumanization and antipathy. That is, this approach allows us to determine whether changes in humanness perceptions (1 and 2) and changes in desirability perceptions (3 and 4) are both associated with increased endorsement of harm. The four separate linear regressions allowed us to measure humanness and desirability changes as orthogonal, testing for effects of each with neither confounded by the other.

Before conducting the regression analyses, assumptions of each of the four individual regression models were checked, and influential scores were identified and removed using Cook's distance.^1^ The final samples for each model were well powered to detect effects of interest in the regression analyses. Full regression statistics for each of the four models, including 95% confidence intervals, are reported in table 3.

1. Is the increase in harm towards targets in the animalistic condition relative to the control predicted by a decrease in uniquely human trait attributions for targets in the animalistic condition relative to the control?

**Table 3.** Full regression statistics for each of the models tested in Study 1.

	*b*	95% CI (lower, upper)	*T*	*R^2^*	*F*	d.f.	*p*
Model 1 (humanness)	−0.034	−0.41, 0.34	−0.18	0.0002	0.03	1197	0.859
Model 2 (humanness)	−0.088	−0.43, 0.25	−0.51	0.001	0.26	1196	0.612
Model 3 (desirability)	−0.819	0.92, −0.72	−15.43	0.55	238.0	1192	<0.001
Model 4 (desirability)	0.750	0.64, 0.86	13.09	0.48	171.23	1189	<0.001

Eight data points fell outside of Cook's distance and one additional data point had a standardized residual greater than 3 and so was identified as an outlier. These nine data points were excluded, resulting in a sample of 199 for the first regression model.

Our first linear regression showed the increase in harm towards targets in the animalistic condition relative to the control was not predicted by a decrease in uniquely human trait attributions to targets in the animalistic condition relative to the control, *p* = 0.859. This model explained less than 0.001% of the variance in relative harm scores.

2. Is the increase in harm towards targets in the animalistic condition relative to the control predicted by an increase in attributions of traits shared with other animals for targets in the animalistic condition relative to the control?

Ten data points fell outside of Cook's distance and were excluded, resulting in a sample of 198 for the second regression model.

Our second simple linear regression showed the increase in harm towards targets in the animalistic condition relative to the control was not predicted by an increase in attributions of traits shared with other animals to targets in the animalistic condition relative to the control, *p* = 0.612. This model explained about 0.001% of the variance in relative harm scores.

3. Is the increase in harm towards targets in the animalistic condition relative to the control predicted by a decrease in socially desirable trait attributions for targets in the animalistic condition relative to the control?

Fourteen data points fell outside of Cook's distance and were excluded, resulting in a sample of 194 for the third regression model.

Our third linear regression showed increased harm towards targets in the animalistic condition relative to the control was significantly associated with decreased desirable attributions to targets in the animalistic condition relative to the control, *p* < 0.001. This model explained approximately 55% of the variance in relative harm scores.

4. Is the increase in harm towards targets in the animalistic condition relative to the control predicted by an increase in socially undesirable trait attributions for targets in the animalistic condition relative to the control?

Seventeen data points fell outside of Cook's distance and were excluded, resulting in a sample of 191 for the fourth regression model.

Our fourth linear regression showed increased harm towards targets in the animalistic condition relative to the control was significantly associated with increased undesirable trait attributions to targets in the animalistic condition relative to the control, *p* < 0.001. This model explained approximately 48% of the variance in relative harm scores.

**Figure 2.**
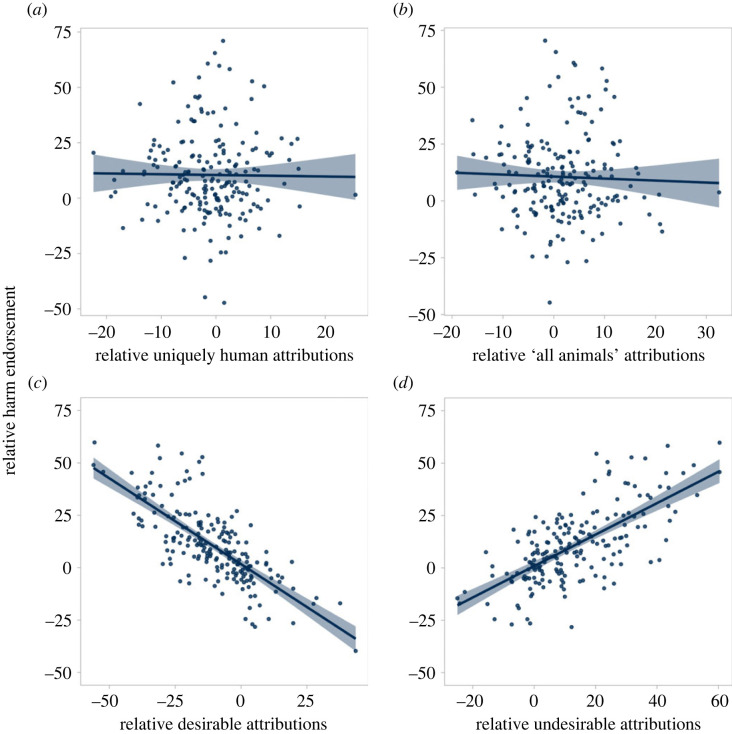
The relationships between trait attributions and harm endorsement in Study 1. Increased harm endorsement towards targets in the animalistic condition relative to the control was predicted by changes in perceptions of social desirability (*c*,*d*, bottom panels) but not by changes in perceptions of humanness (*a*,*b*, top panels).

Increased harm for targets in the animalistic condition relative to the control was predicted by altering perceptions of social desirability but not of humanness (figure 2). Electronic supplementary material, figure S2 shows these relationships for each trait item.

### 2.3. Discussion

Describing a group with animalistic slurs did not lead to trait-based dehumanization of its members compared with a control. Rather, comparing a group to animals changed the extent to which its members were judged to possess socially desirable character traits. Members of the group compared to animals were rated as possessing desirable traits to a lesser extent but undesirable traits to a greater extent than the control, regardless of whether or not these traits were perceived as being unique to humans. Describing a group in animalistic terms increased harm endorsement relative to a control. Crucially, this increased harm was not predicted by changes in the extent to which the group was believed to have uniquely human character traits. Rather, it was predicted by perceptions that members of the target group possessed desirable traits to a lesser extent, and undesirable traits to a greater extent, than the control. We next measured whether this pattern of results would replicate using a different operationalization of dehumanization.

## 3. Study 2: animalistic slurs, emotion attributions and links to harm

In Study 2, we sought to measure whether the results of Study 1 would replicate when employing an alternative characterization of dehumanization based on emotion attribution. According to infrahumanization theory, outgroups are dehumanized to the extent they are denied the experience of uniquely human emotions such as pride, nostalgia and guilt [11,12]. We measured whether describing a novel political group with animalistic slurs changes the extent to which they are viewed as experiencing uniquely human emotions, the extent to which they are viewed as experiencing socially undesirable emotions, or both. We also measured how any change in perception of emotional experience related to harm.

A dehumanization view predicts that reading animalistic slurs will lead participants to view the target group as less human, here shown in the denial of uniquely human emotions. This view further predicts that a dehumanized perception of the target group will explain the connection between animalistic slurs and harm

We predicted that reading animalistic slurs about a group would lead participants to view the target group more negatively, shown in a denial of socially desirable emotions, but increased attribution of socially undesirable emotions. We further predicted that this negative perception of the target group would explain the connection between animalistic slurs and endorsement of harm.

### 3.1. Methods

#### 3.1.1. Participants

Based on the same power analyses described for Study 1, 208 participants completed the study. Participants were again 18 or over, fluent in English, and UK nationals living in the UK. In line with our pre-registration, we excluded and replaced six participants who failed the attention check. Of the final sample, 161 participants identified as female, 44 as male and three as non-binary. Participants were aged from 18 to 74 with a mean age of 38.0 (s.d. = 12.8).

#### 3.1.2. Design

The design and data analysis strategy was as described for Study 1, though with emotion attributions in place of trait attributions.

#### 3.1.3. Materials

##### 3.1.3.1. Experimental manipulation—animalistic and control descriptions

The language manipulation and the harm endorsement scale were exactly as described for Study 1 (table 1 for language manipulation), as was the counterbalancing.

##### 3.1.3.2. Dependent variable 1—emotion attributions

To measure dehumanization as operationalized by infrahumanization theory [11,12], participants rated the extent to which they thought each target group typically experiences a series of 12 different emotions taken directly from related prior work ([31], studies 2–4). The emotions were previously chosen from a large pretest to represent four categories of interest: uniquely human and socially desirable, uniquely human and socially undesirable, shared with other animals and socially desirable and shared with other animals and socially undesirable. In parallel to the traits included in Study 1, the emotion categories took a 2 × 2 structure (humanness: uniquely human/shared with other animals × desirability: socially desirable/socially undesirable). The design allows us to test for subtle dehumanization when the desirability of the emotion terms is controlled for. See electronic supplementary material for more about selection of emotions. Table 4 shows the emotion items included within each condition.

**Table 4.** Emotion items included for each condition in Study 2.

	desirable	undesirable
uniquely human	admiration	contempt
	humility	envy
	optimism	resentment
all animals	contentment	anger
	joy	frustration
	tenderness	irritation

Participants indicated the extent to which they thought members of each group (the MRP and the NLP) typically feel each of the emotions. Emotion attributions were made across two blocks, one for each group condition. For each item, participants indicated their response on a sliding scale from *Not at all* (0) to *Very much so* (100), with the midpoint *Somewhat* (50), though they could not see the numbers. For example, a block could begin:

*Using the sliding scales below, please consider the extent to which you think members of the NLP typically feel each emotion*.

Then, participants would respond to each item, such as ‘Members of the NLP typically feel admiration’ using the unmarked sliding scale. The 12 items within each block were randomized. One attention check per block was included halfway through (e.g. ‘Members of the NLP typically feel: Please indicate “very much so”’).

Following our pre-registered plan, attribution scores for each emotion category for each target group were obtained by calculating the mean of the three emotions within the category for each participant. For example, a participant's attribution of uniquely human desirable emotion for each group was the mean of their ratings for admiration, humility and optimism.

##### 3.1.3.3. Dependent variable 2—harm endorsement

The harm endorsement scale used was exactly as described for Study 1.

#### 3.1.4. Procedure

Participants were informed that the study was designed to help us understand the ways in which people ascribe emotions to different groups of individuals and make behavioural decisions about them. The procedure was then exactly as described for Study 1, though with the emotion attribution scale in place of the trait attribution scale.

### 3.2. Results

#### 3.2.1. The influence of animalistic slurs on emotion attribution

We measured how comparing a target group to animals influenced subsequent emotion attributions for that group. There were four emotion conditions: uniquely human and desirable, uniquely human and undesirable, shared with other animals and desirable, and shared with other animals and undesirable.

A 2 (condition: animalistic/control) × 2 (emotion humanness: uniquely human/shared with other animals) × 2 (emotion desirability: socially desirable/socially undesirable) within-subjects ANOVA measured the effect of animalistic language on emotion attributions. If animalistic slurs lead to representations of the target group as less human, then an interaction between condition and humanness should show ratings of uniquely human emotions to be lower in the animalistic than in the control condition. If animalistic slurs lead to representations of the target group as undesirable in character, then an interaction between condition and social desirability should show ratings of desirable emotions to be lower in the animalistic than in the control condition, and ratings of undesirable emotions to be higher in the animalistic than in the control condition. As noted for Study 1, this design also allows us to detect evidence for both effects operating simultaneously.

There was no main effect of condition, *F*_1,207_ = 001, *p* = 0.974, $\eta_{p}^{2}<0.001$, but there was a main effect of humanness, *F*_1,207_ = 51.06, *p* < 0.001, $\eta_{p}^{2}=0.198$ and of desirability, *F*_1,207_ = 285.01, *p* < 0.001, $\eta_{p}^{2}=0.579$. Ratings were higher overall for emotions shared with other animals than uniquely human emotions, and for undesirable than desirable emotions.

In this study, the three-way interaction between condition, humanness and desirability was not significant, *F*_1,207_ = 0.40, *p* = 0.529, $\eta_{p}^{2}=0.002$.

Measuring whether animalistic slurs alter perceptions of social desirability, there was a significant interaction between condition and desirability, *F*_1,207_ = 78.97, *p* < 0.001, $\eta_{p}^{2}=0.276$. In line with our predictions, ratings were higher for the control condition than the animalistic condition for desirable emotions, but higher for the animalistic than the control condition for undesirable emotions (*ps* < 0.001).

Measuring whether animalistic slurs alter perceptions of humanness, the interaction between condition and humanness was not significant, *F*_1,207_ = 1.06, *p* = 0.305, $\eta_{p}^{2}=0.005$. There were no significant differences between the conditions either for uniquely human emotion attributions or for attributions of emotions shared with other animals.

The theoretically uninteresting interaction between humanness and desirability was significant, *F*_1, 207_ = 56.36, *p* < 0.001, $\eta_{p}^{2}=0.214$. There was no difference in ratings for uniquely human and shared desirable emotions (*p* = 0.561). However, ratings were higher overall for undesirable emotions that were shared with other animals than for uniquely human undesirable emotions (*p* < 0.001). Ratings were higher for undesirable than desirable emotions across both levels of humanness (both *ps* < 0.001).

**Figure 3.**
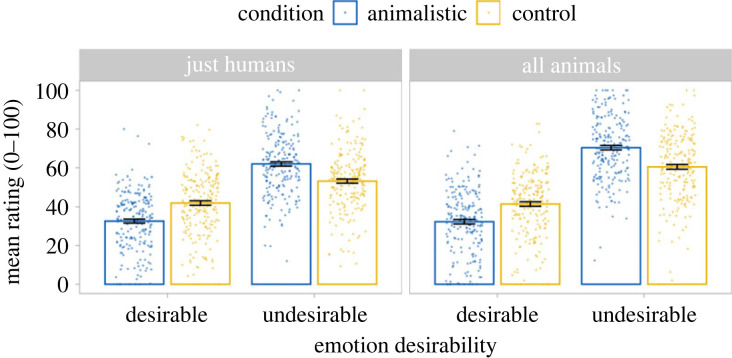
Mean emotion attributions by target group, humanness and desirability conditions for Study 2. Mean ratings were higher for the control than the animalistic condition for desirable emotions but higher for the animalistic condition than the control for undesirable emotions. This was true for uniquely human emotions and for those shared with other species. Error bars represent standard errors.

Animalistic slurs change perceptions of a target's social desirability, but not of their humanness (figure 3). The overall pattern of results held for each of the three items within each emotion category. Electronic supplementary material, figure S3 shows the emotion attributions broken down by items within each condition.

#### 3.2.2. The influence of animalistic slurs on harm endorsement

In line with previous theorizing, participants were more willing to endorse harm against the target group in the animalistic condition (*M* = 50.6, s.e. = 1.56) than in the control condition (*M* = 42.0, s.e. = 1.44), *t*_207_ = 5.97, *p* < 0.001, *d* = 0.41.

#### 3.2.3. The relationship between animalistic slurs, emotion attribution and harm

We tested the mechanism by which comparisons to non-human animals increase a group's risk of being harmed. If animalistic comparisons increase a group's risk of harm through reducing perceptions of their humanity, then we expect an increase in harm endorsement for the animalistic condition relative to the control to be predicted by a decrease in uniquely human emotion attributions and/or an increase in attributions of emotions shared with other animals.

If comparisons to non-human animals lead to an increase in a group's risk of harm through influencing perceptions of their social desirability, then we expect an increase in harm for the animalistic condition relative to the control to be predicted by a decrease in desirable emotion attributions and/or an increase in undesirable emotion attributions.

Four linear regressions tested whether increased endorsement of harm towards targets in the animalistic condition relative to the control was predicted by (1) a decrease in uniquely human emotion attributions; (2) an increase in attributions of emotions shared with other animals; (3) a decrease in desirable emotion attributions; (4) an increase in undesirable emotion attributions.

#### 3.2.4. Creating relative harm endorsement scores (outcome variable)

As for Study 1, to create a relative harm score representing the difference in harm endorsement for the animalistic compared with the control condition, we subtracted each participant's harm endorsement score for the control condition from their harm endorsement score for the animalistic condition.

#### 3.2.5. Creating relative emotion attribution scores (predictor variables)

Similar to Study 1, to create a relative humanness attribution score representing the difference in attribution of uniquely human emotions for the animalistic condition compared with the control, we created the mean of all uniquely human emotions for each participant for each condition, and we then subtracted the mean uniquely human emotions attribution score for the control condition from the mean uniquely human emotions attribution score for animalistic condition. This gave a score showing the difference in humanness attribution between the animalistic and control conditions. Positive numbers here represent a tendency for participants to believe targets compared to animals possess uniquely human emotions to a greater extent than the control, negative numbers indicate the reverse.

We used the same method to create a relative score representing the difference in attribution of emotions shared with other animals for the animalistic condition compared with the control, where positive numbers represent a tendency for participants to believe targets compared to animals possess emotions shared with other animals to a greater extent than the control, and negative numbers indicate the reverse.

We created a relative score representing the difference in attribution of desirable emotions for the animalistic condition compared with the control by creating a mean of the desirable emotions for each participant for each condition, and then subtracting the mean desirable emotion attribution score for the control condition from the mean desirable emotion attribution score for animalistic condition. Positive numbers here represent a tendency for participants to believe targets compared to animals possess desirable emotions to a greater extent than the control, negative numbers indicate the reverse. We used the same method to create a relative score representing the difference in attribution of undesirable emotions for the animalistic condition compared with the control, where positive numbers represent a tendency for participants to believe targets compared to animals possess undesirable emotions to a greater extent than the control, and negative numbers indicate the reverse.

We used these relative scores to test the predictions using four linear regressions.

1. Is the increase in harm for the animalistic condition relative to the control predicted by a decrease in uniquely human emotion attributions for the animalistic condition relative to the control?
2. Is the increase in harm for the animalistic condition relative to the control predicted by an increase in emotion attributions shared with other animals for the animalistic condition relative to the control?
3. Is the increase in harm for the animalistic condition relative to the control predicted by a decrease in desirable emotion attributions for the animalistic condition relative to the control?
4. Is the increase in harm for the animalistic condition relative to the control predicted by an increase in undesirable emotion attributions for the animalistic condition relative to the control?

Note that it is again plausible we could see evidence for both accounts using this approach.

Assumptions of each of the four individual regression models were checked and influential scores were identified and removed using Cook's distance.^2^ The final samples for each model were well powered to detect effects of interest. Full regression statistics are shown in table 5.

1. Is the increase in harm towards targets in the animalistic condition relative to the control predicted by a decrease in uniquely human emotion attributions for targets in the animalistic condition relative to the control?

**Table 5.** Full regression statistics for each of the models tested in Study 2.

	*b*	95% CI (lower, upper)	*T*	*R^2^*	*F*	d.f.	*p*
Model 1 (humanness)	−0.048	−0.37, 0.27	−0.30	0.0005	0.09	1195	0.766
Model 2 (humanness)	0.007	−0.28, 0.29	0.05	0.0001	0.002	1192	0.964
Model 3 (desirability)	−0.647	−0.78, −0.51	−9.36	0.31	87.53	1197	<0.001
Model 4 (desirability)	0.726	0.60, −0.85	11.28	0.40	127.10	1194	<0.001

Eleven data points fell outside of Cook's distance and were excluded, resulting in a sample of 197 for the first regression model.

Our first linear regression showed that the increase in harm towards targets in the animalistic condition relative to the control was not predicted by reduced uniquely human emotion attributions to targets in the animalistic condition relative to the control (*p* = 0.766). This model explained less than 0.001% of the variance in relative harm scores.

2. Is the increase in harm towards targets in the animalistic condition relative to the control predicted by increased attributions of emotions shared with other animals for targets in the animalistic condition relative to the control?

Fourteen data points fell outside of Cook's distance and were excluded, resulting in a sample of 194 for the second regression model.

Our second regression showed the increase in harm towards targets in the animalistic condition relative to the control was not predicted by increased attributions of emotions shared with other animals to targets in the animalistic condition relative to the control, *p* = 0.964. This model explained less than 0.001% of the variance in relative harm scores.

3. Is the increase in harm towards targets in the animalistic condition relative to the control predicted by a decrease in desirable emotion attributions for targets in the animalistic condition relative to the control?

Nine data points fell outside of Cook's distance and were excluded, resulting in a sample of 199 for the third regression model.

Our third linear regression showed increased harm towards targets in the animalistic condition relative to the control was significantly associated with reduced attributions of desirable emotions to targets in the animalistic condition relative to the control, *p* < 0.001. This model explained approximately 31% of the variance in relative harm scores.

4. Is the increase in harm towards targets in the animalistic condition relative to the control predicted by an increase in attributions of undesirable emotions to targets in the animalistic condition relative to the control?

Twelve data points fell outside of Cook's distance and were excluded, resulting in a sample of 196 for the fourth regression model.

Our fourth linear regression showed increased harm towards targets in the animalistic condition relative to the control was significantly associated with increased attributions of undesirable emotions to targets in the animalistic condition relative to the control, *p* < 0.001. This model explained approximately 40% of the variance in relative harm scores.

**Figure 4.**
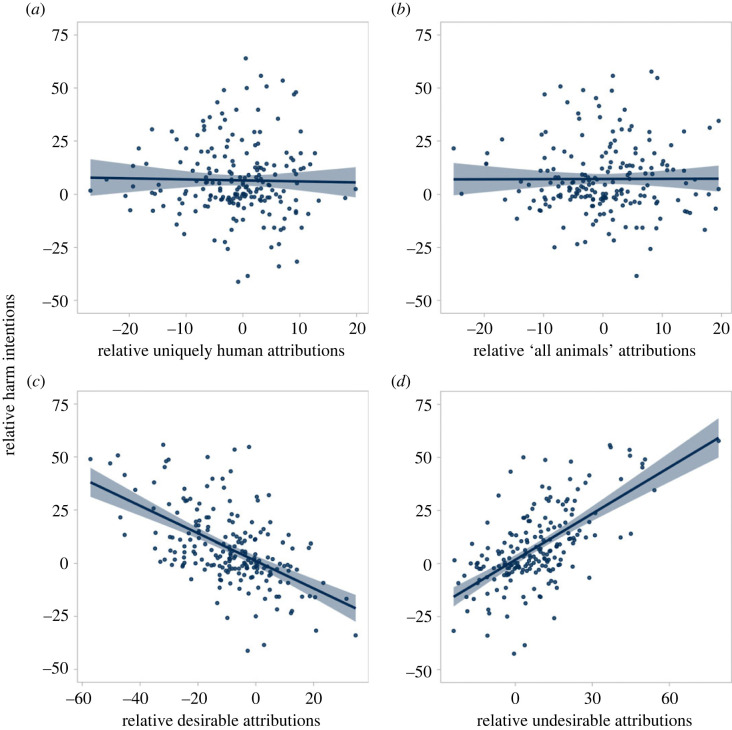
The relationships between emotion attributions and harm endorsement in Study 2. Increased harm endorsement towards targets in the animalistic condition relative to the control was predicted by changes in perceptions of the social desirability of emotional experience (*c*,*d*) but not by changes in perceptions of the humanness of emotional experience (*a*,*b*).

Increased harm directed against targets in the animalistic condition relative to the control was predicted by altering perceptions of social desirability but not of humanness (figure 4). Electronic supplementary material, figure S4 shows these relationships for each emotion item.

### 3.3. Discussion

We replicated our findings from Study 1 using an alternative operationalization of dehumanization [12]. Describing a novel group with animalistic slurs did not lead to perceptions of its members as experiencing uniquely human emotions to a lesser extent. Rather, when described with animalistic slurs the target group were rated as experiencing desirable emotions to a lesser extent but undesirable emotions to a greater extent than the control, regardless of whether or not the emotions were perceived to be unique to humans. As in Study 1, reading animalistic slurs increased endorsement of harm towards the target group. This increased endorsement of harm was predicted by perceptions that the target group experienced desirable emotions to a lesser extent and undesirable emotions to a greater extent than the control, not by differential attributions of humanness.

## 4. General discussion

Propaganda and hate speech are replete with references to target groups as dangerous and disgusting animals. Whereas several researchers argue that these animalistic slurs can be intended literally [3,4,13,34], others maintain that they are often metaphorical—reflecting deep antipathy towards the target group rather than a literal belief that the group are rats, snakes or cockroaches [19,21,22].

Despite receiving theoretical attention across history, ethics, philosophy, sociology and social psychology, the ways in which animalistic slurs influence the attitudes and behaviours of its intended audience has not yet received systematic empirical attention. Across two studies, we examined the impact of animalistic slurs on the cultural transmission of beliefs about novel political groups, along with how these beliefs motivate intergroup harm. In line with previous findings [26,27], we show that the addition of animalistic slurs to otherwise matched sentences increases endorsement of harm to an outgroup. Examining the effects of animalistic slurs on attributions of character traits and emotions, we found that animalistic slurs did not lead to reduced attributions of uniquely human character traits (Study 1) or emotions (Study 2). Instead, across both studies, target groups described with animalistic slurs were perceived as possessing desirable qualities to a lesser extent and undesirable qualities to a greater extent.

The pattern of results differed subtly between studies 1 and 2. In Study 1, there was a three-way interaction between condition, trait humanness and trait desirability, When broken down into simple comparisons, this significant interaction showed that targets described in animalistic terms were attributed desirable traits to a lesser extent and undesirable traits to a greater extent than the control, both for uniquely human terms and ones shared with other animals. In Study 2, the three-way interaction was not significant. Importantly, there was a significant two-way interaction between condition and emotion desirability, suggesting again that targets described using animalistic language were attributed desirable emotions to a lesser extent and undesirable emotions to a greater extent than the control. There was this time no significant interaction between condition and humanness, suggesting that animalistic language did not affect the extent to which targets were attributed uniquely human emotions. Taken together, both studies suggest that rather than animalistic slurs inducing dehumanizing beliefs in an audience (as operationalized by two prominent accounts of dehumanization from social psychology; [9,12]), animalistic slurs instead induce beliefs about socially undesirable characteristics, without also affecting beliefs about human characteristics.

Further supporting our central findings, in both studies, the increased harm endorsement to the target group brought about by the animalistic slurs was not predicted by perceptions that its members possessed uniquely human attributes to a lesser extent. Instead, increased endorsement of harm was predicted by perceptions that the target group possessed desirable attributes to a lesser extent and undesirable attributes to a greater extent.

It is crucial to emphasize that we are not seeking to provide an exhaustive account of how animalistic slurs lead to harm. We focused on two different models of dehumanization—the dual model [9] and infrahumanization theory [12]. It may well be that animalistic slurs lead to dehumanization as operationalized in other ways [13]. For example, it is entirely plausible that hearing animalistic slurs increases the probability that listeners use animalistic references more often in their own speech [8].

In this work, we focused on how animalistic slurs influence attitudes towards novel political groups, and we focused on group-level differences across our two experimental conditions. However, some work has suggested important individual differences in the propensity to dehumanize others, such as political ideology and social status [35]. It will be interesting for future work to examine whether effects of animalistic slurs on intergroup attitudes and harm endorsement vary according to factors such as ingroup identification, political ideology or social dominance orientation (e.g. [36]).

Our measures of harm were procedural rather than relating to the endorsement of physical violence. While our work provides a basis for understanding this complex topic, future work could include measures of other types of harm endorsement such as physical harm along with incorporating, where ethically possible, behavioural measures of harm. Such work could help provide a bridge between laboratory-based research on subtle dehumanization, and philosophical and sociological research on dehumanization and extreme intergroup harm.

It is also important to acknowledge that labelling a group of people as apes, dogs or cockroaches can be intended to degrade, shame and humiliate the target group themselves. This kind of language is likely to damage the psychological wellbeing of targets independently of the attitudes and behaviours it evokes in others, and may indeed feel dehumanizing [32]. Further understanding the effects of animalistic slurs on the cognition and wellbeing of targets is an important direction for future research.

We recognize that not all animal comparisons are equally offensive. Some animal comparisons may be more deeply offensive than others, and different animalistic comparisons may evoke different types of trait representations. For example, whereas referring to a group as ape-like may evoke stereotypes of stupidity, referring to a group as snake-like may evoke stereotypes of dishonesty and betrayal [22]. The roles of social context and of group stereotypes are important for more fully understanding how animal comparisons influence intergroup attitudes and behaviour. Another important direction for future research will be to understand how the impact of animalistic slurs compares with the impact of other negative descriptors found in propaganda, such as references to the target group as criminals, traitors and murderers [19,21,22]. It will also be important to understand how superficially desirable descriptions, such as referring to a group as clever or intellectual, can be used to enhance the extent to which audiences perceive them as a threat.

In this work, we examined the causal role of dehumanizing language in influencing beliefs about others and motivations for harm. Some thinkers have suggested that the role of dehumanizing language in violence may be better characterized as reinforcing social norms surrounding ongoing violence and justifying harms that have already been committed [1]. The role of animalistic slurs in reinforcing social norms at the time of harm, and as serving as *post hoc* justification for historical harm, are also important areas for further study.

Understanding the specific ways in which animalistic slurs, commonly found in propaganda and hate speech, influence the cultural transmission of beliefs about others has important implications. Given historical links between comparisons to non-human animals and intergroup harm, along with the rising prevalence of dehumanizing hate speech online [37–39], accurately understanding its societal impact on attitudes and behaviours is crucial for learning how to challenge it. Taken together, our studies suggest that negative animal comparisons function as metaphors that evoke beliefs about targets as antisocial and socially undesirable. These findings provide a novel perspective on the function of non-human animal comparisons within the study of dehumanization and intergroup bias.

## Data Availability

All studies reported in this article were pre-registered and the data is available open access. Link to pre-registration documents and raw data for each study can be found at: https://osf.io/4pvs3/. Electronic supplementary material is available for this article [40].

## Footnotes

1 While we remove influential scores falling outside of Cook's distance in line with standard practice and our pre-registration, this does not change the overall pattern of significance in our results.

2 As for Study 1, while we remove influential scores falling outside of Cook's distance in line with standard practice and our pre-registration, this does not change the overall pattern of significance in our results.
